# Supplementary material for: Expression of Saccharomyces cerevisiae RER2 Gene Encoding Cis-Prenyltransferase in Trichoderma atroviride Increases the Activity of Secretory Hydrolases and Enhances Antimicrobial Features
Source: J Fungi (Basel). 2022 Dec 26;9(1):38. doi: 10.3390/jof9010038 (PMC9860738; doi:10.3390/jof9010038)
Supplement: Supplementary file 1 [file jof-09-00038-s001.zip › jof-2100793-supplementary.pdf]

Table S1. Primers used in this studies

Primer name	5′- sequence -3′	Amplicon size bp	Purpose
Rer2F_c	CCTATACGGATGGCATGGATTT	105	Primers homologous to the *RER2* gene from *S. cerevisiae*
Rer2R_c	CCCATTGGTTTCCTCGTCATA		
Rer2TaF	TCCCATACACATCGAGAGCAG	198	Primers homologous to the *rer2* gene from *T. atroviride*
Rer2TaR	TTGCCCTCATACTCGCCATC		
Nus1_F	CCGCGAGATATTTCCCAAGA	97	Primers homologous to the *nus1* gene from *T. atroviride*
Nus1_R	GCTCGACGTAAGGAGTGAATAG		
FActTa	AACCGTGAGAAGATGACCCAG	198	Primers homologous to the *act1* gene from *T. atroviride*
RActTa	CCATGTCCACACGAGCAATG		

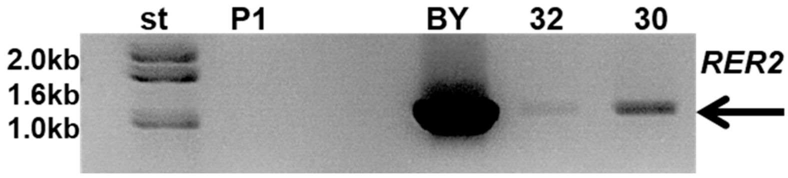

Figure S1. The presence of the yeast *RER2* gene in the genome of the transformants analyzed by PCR
As a positive and negative control the PCR reactions were performed on the templates of DNA from *S. cerevisiae* (BY) and untransformed *T. atroviride* (P1), respectively.
32 and 30 represent PCR products obtained on the template of DNA from RER32/11 and RER30/11 transformants, respectively.
